# Uterine Polypsus Treated by Injections of the Perchloride of Iron

**Published:** 1860-05

**Authors:** 


*Uterine Polypus treated by Injections of the Perchloride of Iron.*—Put her upon an energetic course of treatment—tonics and astringent injections, such as acetate of lead, tannin, sulph. zinc, &c. During the treatment we often introduced into the vagina portions of solid ice; also, bits of alum, enveloped in lint, were introduced into the os uteri. Notwithstanding this course of treatment, she continued, with but occasional respite from the hæmorhage, for between five and six days after we saw her.

Becoming convinced that there must be some cause at the bottom of all this, other than the supposed "change of life," which, as yet, we had not ascertained, we made as thorough an uterine examination as the condition of the uterus would allow, and detected a soft, fleshy substance—pendulous, apparently, from the fundus uteri. As the neck of the uterus remained rigid, and further dilatation was not practicable, ergot was given in small, repeated doses, as well to arrest the bleeding, as to expel, if possible, what seemed to be the cause of the trouble. Having continued the ergot for some ten or fourteen hours, with ice and astringent injections, and the hæmorrhage continuing—sometimes after a partial cessation, returning in alarming quantities—all our previous efforts to arrest it having failed, we injected the muriated tincture of iron, diluted in one-third mucilage gum-arabic, into the os—continuing the opium tonic mixture, and ergot during the night.

June 25th, A. M.—found the patient more comfortable—the os so dilated that we introduced the hand into the uterus sufficient to enable us to remove several small pieces of semi-consistent, hepatized floculi, with some well-defined fibrinous substance, resembling, in part of its formation, the surloin portion of beef, and quite strongly attached to the fundus of the uterus—somewhat larger in its body than a hen's egg, with a vermiform portion extending downward.

The dilatable condition of the uterus at this time, and the ease with which we grasped the tumor, enabled us to remove it without the use of instruments.

During the removal of this body there was considerable hæmorrhage, which was soon arrested by cold injections of alum-water.

It may seem to some that a solution so strong of muriat. tinct. ferri was rather a harsh application, and uncalled for under the premises. The urgency of the case, and the failure of the previous treatment to arrest the discharge, prompted us to resort to this treatment. And then even with the tinct. ferri mur., in its full strength, we are of the opinion that the lubricating secretion thrown out from the mouth of the uterus, would soon form a shield around even this escharotic, and prevent injury to the parts.

